# Rare presentation of primary synovial chondrosarcoma arising in the shoulder: a case report

**DOI:** 10.1007/s00256-024-04811-3

**Published:** 2024-10-08

**Authors:** Kengo Kawaguchi, Makoto Endo, Koji Sagiyama, Akira Maekawa, Akira Nabeshima, Toshifumi Fujiwara, Yoshinao Oda, Yasuharu Nakashima

**Affiliations:** 1 Department of Orthopaedic Surgery, Graduate School of Medical Sciences, Kyushu University, 3-1-1, Maidashi, Higashi-Ku, Fukuoka, Japan; 2 Department of Anatomic Pathology, Graduate School of Medical Sciences, Kyushu University, 3-1-1, Maidashi, Higashi-Ku, Fukuoka, Japan; 3 Department of Health Care Administration and Management, Graduate School of Medical Sciences, Kyushu University, 3-1-1, Maidashi, Higashi-Ku, Fukuoka, Japan; 4 Department of Clinical Radiology, Graduate School of Medical Sciences, Kyushu University, 3-1-1, Maidashi, Higashi-Ku, Fukuoka, Japan; 5 Department of Orthopaedic Surgery, Kyushu Rosai Hospital, 1-1, Sonekitamachi, Kokuraminami-Ku, Kitakyushu, Japan

**Keywords:** Synovial chondrosarcoma, Intra-articular tumor, Trans-articular tumor

## Abstract

Synovial chondrosarcoma (CHS) is a rare malignant tumor arising from the synovial tissue and is often associated with synovial chondromatosis. Herein, we present a unique case of primary synovial CHS in the shoulder joint without evidence of synovial chondromatosis. A 60-year-old man presented to our hospital with a complain of left shoulder pain that persisted for 6 years, which was initially misdiagnosed as synovitis. Radiography revealed an osteolytic lesion involving the humerus and the scapula. Histologically, the tumor exhibited features of grade 2 synovial CHS, infiltrating the trabecular bones and intra-articular space. Wide resection led to a 9-year recurrence-free survival. This case underscores the challenges in diagnosing and managing synovial CHS, particularly in cases with atypical presentations lacking synovial chondromatosis, necessitating careful follow-up and adequate surgical intervention.

## Introduction

Chondrosarcoma (CHS) is a malignant tumor composed of hyaline cartilage matrix and chondroid cells [1]. CHS can form de novo as a primary lesion or result from the malignant transformation of preexisting cartilage lesions or benign conditions, such as enchondroma, osteochondroma, and synovial chondromatosis. CHS arising from pre-existing synovial chondromatosis or de novo in synovial tissue has been called synovial CHS [2, 3]. Synovial CHS without evidence of synovial chondromatosis is particularly rare, with only a few histologically proven cases reported since 1991 [3–6]. Synovial CHS is most prevalent in the knee (consistent with the epidemiology of synovial chondromatosis), followed by the hip, and relatively rare in the shoulder joint [2, 3, 6, 7].

Here, we present a rare case of primary synovial CHS of the shoulder joint that lacked synovial chondromatosis and a review of its clinical, radiological, and histological features.

### Case report

A 60-year-old man presented to our hospital with left shoulder pain that persisted for 6 years. A palpable firm mass was found on the left shoulder. External and internal rotations of the shoulder joint were limited compared with those on the unaffected side. All other clinical findings, past medical history, family history, and laboratory evaluations were unremarkable.

**Fig. 1:**
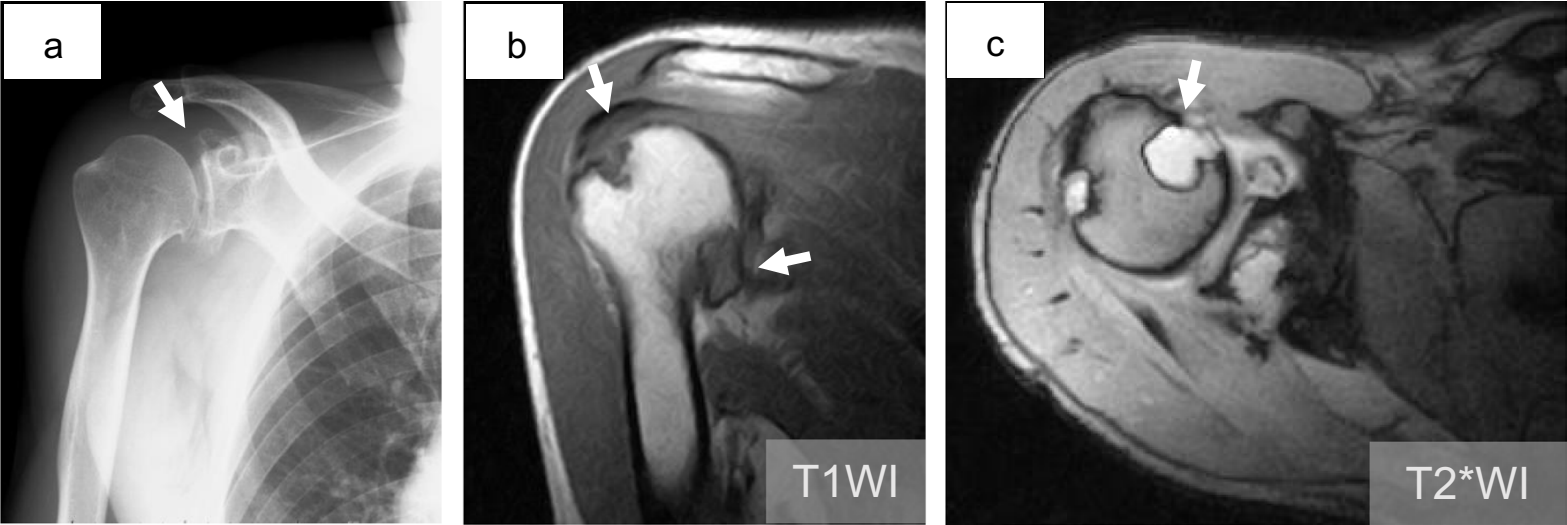
Radiography and MRI taken 6 years prior to diagnosis. **a** Radiography reveals a expanding osteolytic lesion in the glenoid of the scapula (arrow). **b, c** MRI showed intra-articular masses with iso-intensity on T1WI and high-intensity on T2*WI. These masses caused erosion of the humeral head (arrows)

Six years prior to visiting our hospital, he consulted a neighborhood clinic. Radiography images taken at that time showed the cortex of the humerus and scapular glenoid irregularities in an osteolytic lesion (Fig. 1a). On magnetic resonance imaging (MRI), uniform masses exhibiting isointensity on T1-weighted images (WI) and high intensity on T2*WI were identified within the capsule of the shoulder joint and the surrounding scapular tissue (Fig. 1b, c). Intraosseous lesions of the intensity same as that of the extraosseous lesions were observed in the humerus and scapula. A physician at the clinic suspected synovitis based on observations. The patient was treated conservatively with rest and non-steroidal anti-inflammatory drugs. Follow-up radiographic examinations were not performed.

**Fig. 2:**
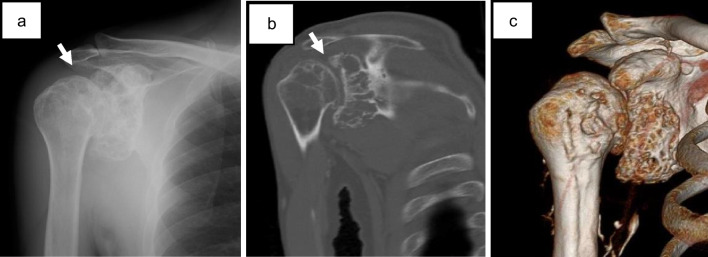
Radiography and CT images taken in our hospital. **a** Radiography reveals irregular osteolytic lesions with sclerotic changes in both the scapular and proximal humerus (arrow). **b, c** CT images show intramedullary osteolytic lesions with focal cortical destruction (arrow)

**Fig. 3:**
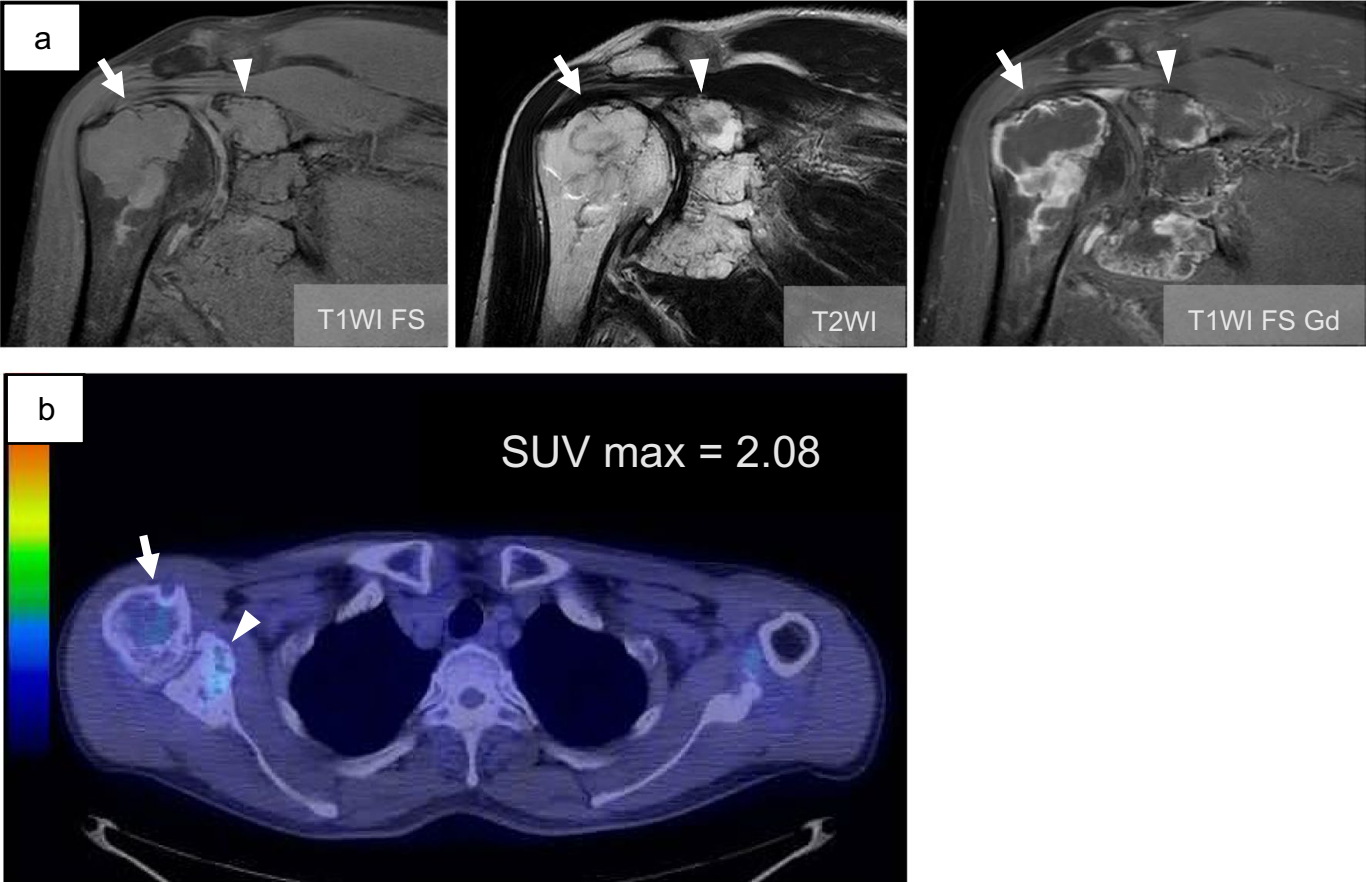
MRI and PET-CT image. **a** An MRI scan reveals the presence of multi-lobulated intramedullary tumors exhibiting iso-intensity on T1WI and homogeneous high signal intensity on T2WI with peripheral gadolinium enhancement in both the scapula (arrowhead) and proximal humerus (arrow). **b** PET-CT scan reveals SUV max value 2.08 hot spot in both scapula (arrowhead) and humeral head (arrow)

Radiography at our hospital revealed an expanding osteolytic lesion in the glenoid of the scapula (Fig. 2a). Computed tomography (CT) revealed intramedullary osteolytic lesions and focal cortical destruction without calcification (Fig. 2b, c). An MRI scan revealed the presence of multilobulated intramedullary tumors, which exhibited isointensity on T1WI and homogeneous high signal intensity with peripheral and septal gadolinium enhancement in both the scapula and proximal humerus (Fig. 3a). 18F-fluorodeoxyglucose-positron emission tomography (FDG-PET) revealed minimal FDG uptake in the humerus and scapula (maximum standardized uptake value [SUV_max_] = 2.08; Fig. 3b). No distant metastases were found on the PET or chest CT images.

**Fig. 4:**
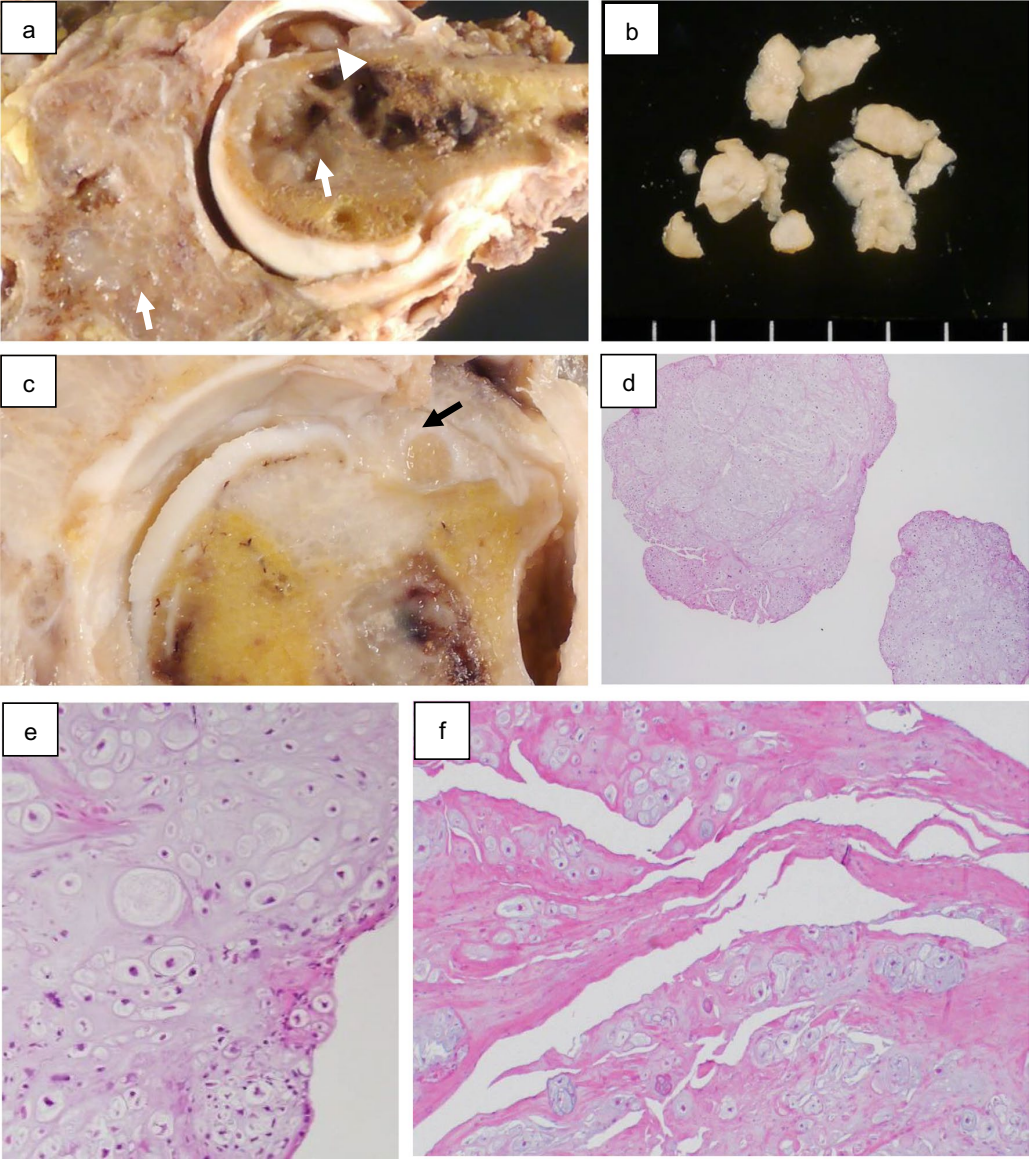
Gross image of the cut surface and histopathology of intra-articular free-bodies and synovium. **a, b** The resected specimen shows intramedullary myxoid grayish-white masses (**a**, arrows) and intra-articular nodules (**a**, arrowhead, **b**). **c** The articular capsule in the greater tuberosity is penetrated by the tumor tissue (arrow). **d, e** Intra-articular nodules showing a vague lobular structure of hyaline cartilage with occasional binucleated chondrocytes. Some chondrocytes have pleomorphic nuclei. **f** Cartilaginous tumor cells infiltrate the collagenous joint capsular tissue

Open biopsy was performed. A specimen from the scapula showed fragments of mildly atypical cartilaginous tissue. The biopsy revealed a cartilaginous tumor, suggestive of CHS. Subsequently, wide resection was performed. The scapula and humerus were resected *en bloc* with the extracapsule technique. Gross findings showed that the tumors were present in both the scapula and humerus and were composed of lesions that were soft and gelatinous in texture and whitish-gray in color (Fig. 4a). The numerous free cartilaginous bodies were observed in the shoulder joint (Fig. 4a, b). Moreover, the capsule on the greater tuberosity of the humerus was penetrated by tumor cells (Fig. 4c).

**Fig. 5:**
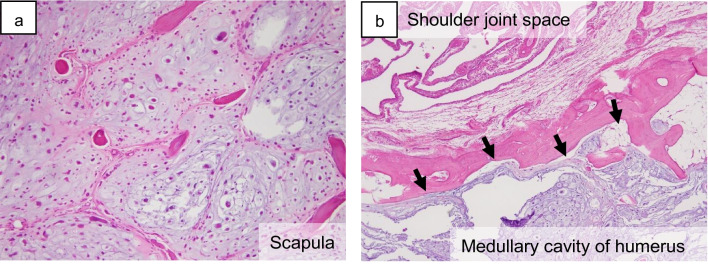
Histopathology of lesions localized on the scapula and humerus. **a** In the scapula, the tumor tissue demonstrated multinodular proliferation of atypical chondrocytes with prominent nuclear pleomorphism, accompanied by entrapped cancellous bone. **b** The medullary cavity of the proximal humerus beneath the cortex was filled with cartilaginous tumor tissue (arrows). The permeative pattern in the scapula was also seen in the proximal humerus (not shown)

Histologically, the intra-articular-free bodies showed a vague lobular structure of the hyaline cartilage, with occasional binucleated chondrocytes and some chondrocytes with pleomorphic nuclei (Fig. 4d–f). Atypical chondrogenic tumor cells were also present within the synovium (Fig. 4g). In both the humerus and scapula, tumor cells had permeated the trabecular bones in both the humerus and scapula (Fig. 5). High-power view showed the proliferation of atypical chondroid cells with hyperchromatic nuclei accompanied by a chondroid or myxoid matrix. Tumor invasion was observed on the articular surface, capsule, and labrum. The final diagnosis was grade 2 synovial CHS. Neither synovial chondromatosis nor benign cartilaginous components were detected in any of the specimens. Additionally, fluorescence in situ hybridization did not detect any gene rearrangement of fibronectin 1 (*FN1*), which is frequently observed in synovial chondromatosis and CHS arising from it [8, 9]. The patient has been recurrence- and metastasis-free for 9 years after surgery.

## Discussion

Herein, we report a unique case of primary synovial CHS of the shoulder joint. While synovial CHS secondary to synovial chondromatosis can disseminate within joints [10, 11], this case showed no evidence of synovial chondromatosis.

It has been reported that around 60 cases of synovial CHS have been documented, which are classified into the following three categories based on their clinical presentation: (i) synovial CHS present in patients with a history of synovial chondromatosis and without areas of chondromatosis at the time of diagnosis of malignancy; (ii) synovial CHS presenting in patients without a history of synovial chondromatosis and with histological evidence of concomitant chondromatosis at the first diagnosis of synovial CHS; and (iii) synovial CHS presenting in patients without a history or concurrent histological evidence of synovial chondromatosis [2–7, 11–23]. The present case involved the third type, with only four similar cases reported in the literature [3, 4, 6]. Although the possibility that all preexisting synovial chondromatosis lesions could have been replaced by those of CHS cannot be completely ruled out, it is presumed to be unlikely.

Regarding clinical imaging studies of synovial CHS, radiography helps observe intra-articular calcifications and an irregular bone cortex [24]. However, benign lesions such as synovial chondromatosis may show similar findings [22]. Although CT is helpful in identifying minute soft tissue calcifications and mild localized bone erosions that may not be visible on radiography, MRI can more clearly show the anatomical details and extent of the lesion. In this case, it was difficult to diagnose a malignant neoplasm, including synovial CHS, at the first visit to the primary physician because the deformities of the glenoid and humeral head resembled osteophytes and bone erosions at the insertion of ligaments. While a rapid worsening of symptoms and an increase in tumor size are generally considered signs of malignancy, surgeons should consider the slow progress of some types of malignant tumors, as in this case.

Pathologically, synovial CHS presents with findings similar to those of central CHS. The distinction from synovial chondromatosis, which is often difficult to determine in clinical imaging studies, is based on the loss of clustering of chondrocytes, the presence of myxoid change of the stroma, hypercellularity in the periphery, peripheral spindling of chondrocytes, necrosis, and permeation of trabecular bone in a “filling-up” pattern [2]. As the present condition met these criteria, we diagnosed synovial CHS and concluded that it was equivalent to grade 2 according to the World Health Organization classification [1]. It should also be noted that the patient had numerous small intra-articular free bodies which exhibited the features of grade 2 CHS. Although central, peripheral, and periosteal CHSs are known to extend extraosseously [1], there are no reports of free bodies within invaded joints in these tumors. Therefore, we considered the possibility that this case involved primary synovial CHS.

Comparison of the clinical behavior of other CHS subtypes with that of synovial CHS is difficult because of a limited number of reported cases. Wide resection and limb salvage in synovial CHS are more complicated than those in conventional CHS because the affected lesions exist on both sides of the joint. It is reported that the amputation above the affected joint was performed for approximately 60% of patients with synovial CHS, and local recurrence occurred in up to half of cases [18]. The patient underwent extensive extracapsular surgical resection with adequate margins and survived without adjuvant therapy.

In conclusion, we report a case of synovial CHS of the shoulder with slow progression. This was an unusual case of a tumor that straddled a joint and disseminated lesions within the joint that lacked findings suggestive of synovial chondromatosis. Although the details of synovial CHS are primarily unknown, clinicians should follow-up on cases that are difficult to diagnose with imaging and are atypical for benign diseases.
